# Remotely Triggered Locomotion of Hydrogel Mag-bots in Confined Spaces

**DOI:** 10.1038/s41598-017-16265-w

**Published:** 2017-11-23

**Authors:** Tong Shen, Marti Garriga Font, Sukwon Jung, Millicent L. Gabriel, Mark P. Stoykovich, Franck J. Vernerey

**Affiliations:** 1 Mechanical Engineering, University of Colorado Boulder, 427 UCB Boulder, USA; 2 Chemical and Biological Engineering, University of Colorado Boulder, 596 UCB Boulder, USA

## Abstract

In this study, soft hydrogel crawlers with remote magnetic-responsive motility in confined spaces have been developed. Inspired by the motion of maggots, the hydrogel crawlers can reversibly contract and elongate their body controlled by repeatedly switching on/off an alternating magnetic field. Based on the cyclic deformation, the hydrogel crawlers can move peristaltically in a confined space that is coated with asymmetric micro-patterns. The dependence of the hydrogel motility on the pattern structures and lubrication is characterized using experimental measurements. Such a hydrogel system pioneers the study of active motile systems in porous media and has the potential to impact the fields of targeted drug delivery and active actuators.

## Introduction

Synthetic motile particles made by soft materials have been of great interest to the field of drug delivery^1^, stimuli-responsive systems^2^ and bio-inspired robots^3^. In nature, motion is ubiquitous from the nano to the macro scales. One essential condition for an organism to achieve locomotion is to break its front-back symmetry or to take advantage of existing asymmetries in its environment^4^. For example, at the micro-scale, amoeba^5^ and leukocytes^6^ protrude their leading edge as an anchor and use the differences in adhesion between the head and rear of the cell to contract their body for locomotion. At the macro-scale, maggots and snakes^7^ take advantage of the asymmetric friction generated by the patterns of their skin to achieve directional motion.

These natural systems have inspired scientists to use the idea of symmetry breaking to develop synthetic motile systems. In general, these systems can be divided into two main categories: swimmers and walkers. The first kind, synthetic swimmers, are micro-robots or colloidal particles that are able to propel themselves by interacting with aqueous solution via asymmetrically deforming their body^8^, or via mechanisms such as phoresis^9–11^ and the Marangoni effect^12^. These systems can potentially be used for the study of source targeting, directional control and swarming^12,13^. However, their motion is restricted to environments with low Reynold’s numbers, and their efficiency may be greatly affected by convection or other perturbations in the surrounding fluid.

The second kind of synthetic mobile system, walkers, are usually made of soft materials and are able to achieve locomotion by interacting with the substrate and move using frictional forces. Compared to swimmers, walkers are able to function in a wider range of sizes and environments. One of the well-known systems is the motion of a Leidenfrost droplet on a ratcheted substrate propelled by asymmetries in the vapor pressure^14,15^. Polymeric materials have been extensively used in these systems as well, due to their deformability, allowing them to move through narrow pores^16–18^ and their ability to respond to various external stimuli including pH gradients, temperature, and electric fields^19,20^. Mahadevan *et al*.^21^ took advantage of the softness of a cylindrical hydrogel particle and simulated the motion of some limbless terrestrial animals using an external vibration source. Lee *et al*.^22^. developed a hygromorphic actuator that actively “rolls” on a ratchet plate in response to the changes in the relative humidity. Besides, the walking motion of an “arch-shaped” hydrogel by cyclic bending/stretching on ratchets has also been achieved using various stimuli, such as chemical oscillators^23^, electric fields^24–26^ or the change of lights in the environment^27^. While these systems have shown motility, their motion needs to be locally stimulated or is restricted to certain fluid environments (e.g., in electrolyte solutions).

While most of these particles are able to migrate on a plane, we are here interested in another type of environment: the interstitial space of 3D porous media including biological tissues, foams, fibrous and granular materials to cite a few. Motion achieved in this class of media has important applications in drug delivery or cargo transport for material repair. In order to achieve motion in such media, there are, however, two key challenges to address: first the particle must overcome the effect of confinement created by the surrounding material and second, motion must be triggered remotely since the particle cannot be accessed when located deep inside a porous medium. For this class of locomotion, telescoping peristaltic motion has been commonly observed among organisms such as maggots^28,29^, and earthworms^30^. These organisms generate an elongation wave along their body and establish directional frictional contact with the confined surrounding using the ratcheted structure on their skin. In synthetic systems, such peristaltic deformation has been mimicked by a Belousov-Zhabotinsky reaction that periodically triggers the swelling/deswelling of a hydrogel particle^31,32^. This approach however did not produce enough deformation to enable efficient motion^33^. Yeghizarian and colleagues^4^ later achieved motion with a hydrogel in a confined tube by manually propagating volumetric phase transitions as a peristaltic wave along its body. In this approach, the hydrogel exhibited sustained motion similar to that of an earthworm, however, this system required manual and localized actuation and thus its potential may in practice be limited. The availability of efficient, remote controlled motile systems for porous media therefore remains an obstacle to the development of soft actuators and drug delivery systems. In this study, we use inspiration from the crawling mechanics of maggots and larvae to develop a millimeter-size hydrogel particle (referred to as Mag-bot for “Magnetically Actuated Gel Bot”) that can crawl between two confined plates and that is powered by a remote magnetic field. While the proof of concept behind such as system was demonstrated in earlier work at the centimeter scale^34^, the contributions of this work is three folds: (a) the mag-bot is magnetically actuated and can therefore be powered from a distance, (b) locomotion is enabled via the fabrication of a micron-sized scale pattern that has the potential of being transferred onto the mag-bot’s body in the future and (c) the system size is reduced to sub-millimeter scale, improving its potential for biomedical applications. Taken together, these contributions demonstrate the actuation of sub-millimeter soft crawler by a remote power source, potentially opening the doors to the simultaneous actuation and motion of a population of micro-crawlers (or mag-bots) in porous media.

## Fabrication and performance of a Mag-bot

**Figure 1:**
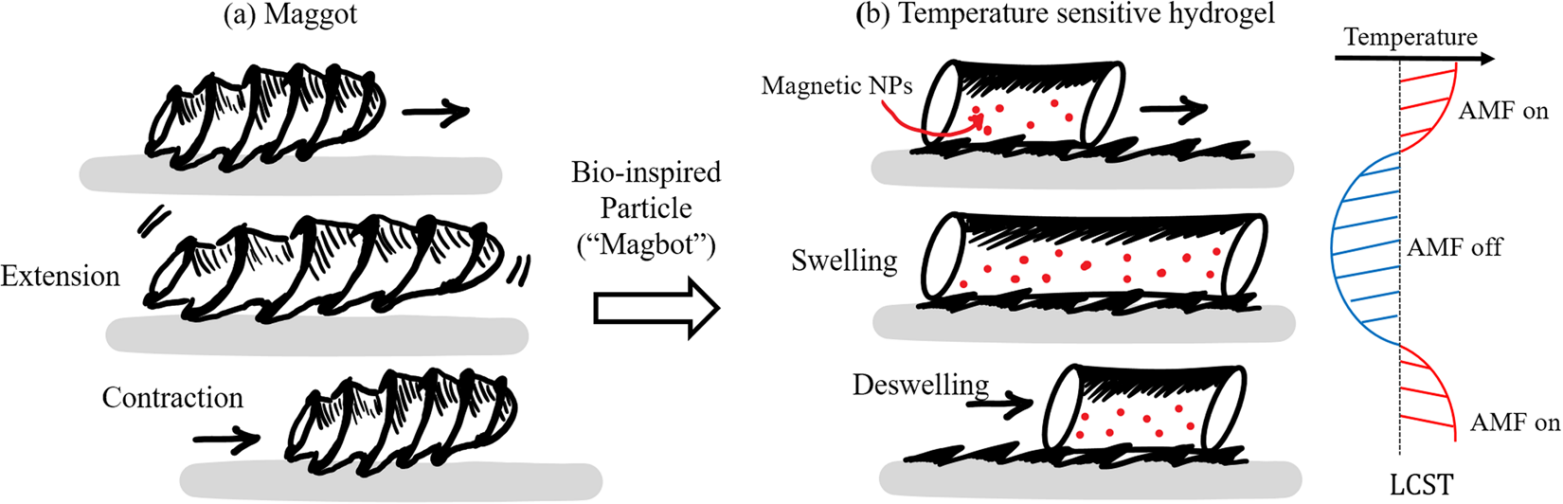
Particle motion and bio-inspiration (**a**) Schematic showing the crawling motion of a common maggot. Locomotion relies on two features: 1. actuation, via the periodic extension-contraction of the maggot’s body and 2. Symmetry breaking resulting from the ratchet-like patterns on its skin. (**b**) Active particles can be made following similar mechanisms when fabricated with a temperature-sensitive NiPAAm hydrogel loaded with magnetic nano-particles. Motion can be generated by remotely heating the particle with a magnetic field, inducing a swelling-deswelling motion, reminiscent of that of maggots. Symmetry breaking can then be achieved by coating the substrate with scale-like features.

In this study, the mag-bot consists of a hydrogel particle made of a crosslinked, thermo-sensitive poly-N-isoproprylacrylamide (PNIPAAm) network and *Fe*_3_ *O*_4_ superparamagnetic nanoparticles. The hydrogel can reversibly contract and expand in response to temperature changes induced by the oscillation of an alternating magnetic field. A directional crawling motion is achieved by allowing the confined hydrogel particle to interact with surfaces coated with ratchet-shaped micropatterns. One key feature of this system is the application of magnetic field that enables remotely controlled deformation of the hydrogel. For applications such as targeted drug delivery or bioimaging, many existing strategies of stimulation are inappropriate due to limitation in penetration depth (e.g., light) or potential safety issues (e.g., high electric fields)^35^. Magnetic field is ideal due to its safety and easiness to control, and have been intensively studied for clinical application such as hyperthermia^36,37^. Another key feature of the system is the application of anisotropic micro-patterns that drives directional motion of the hydrogel particle. Unlike the “hooking” mechanism of “walking” polymer sheets^23,26^, the hydrogel particle crawls by establishing differential frictional interactions with the anisotropic geometry provided by the ratchet-shaped microstructures. This mechanism of motion resembles that of earthworms and maggots and has the potential to enable remote motion in 3D porous media (Fig. 1).

**Figure 2:**
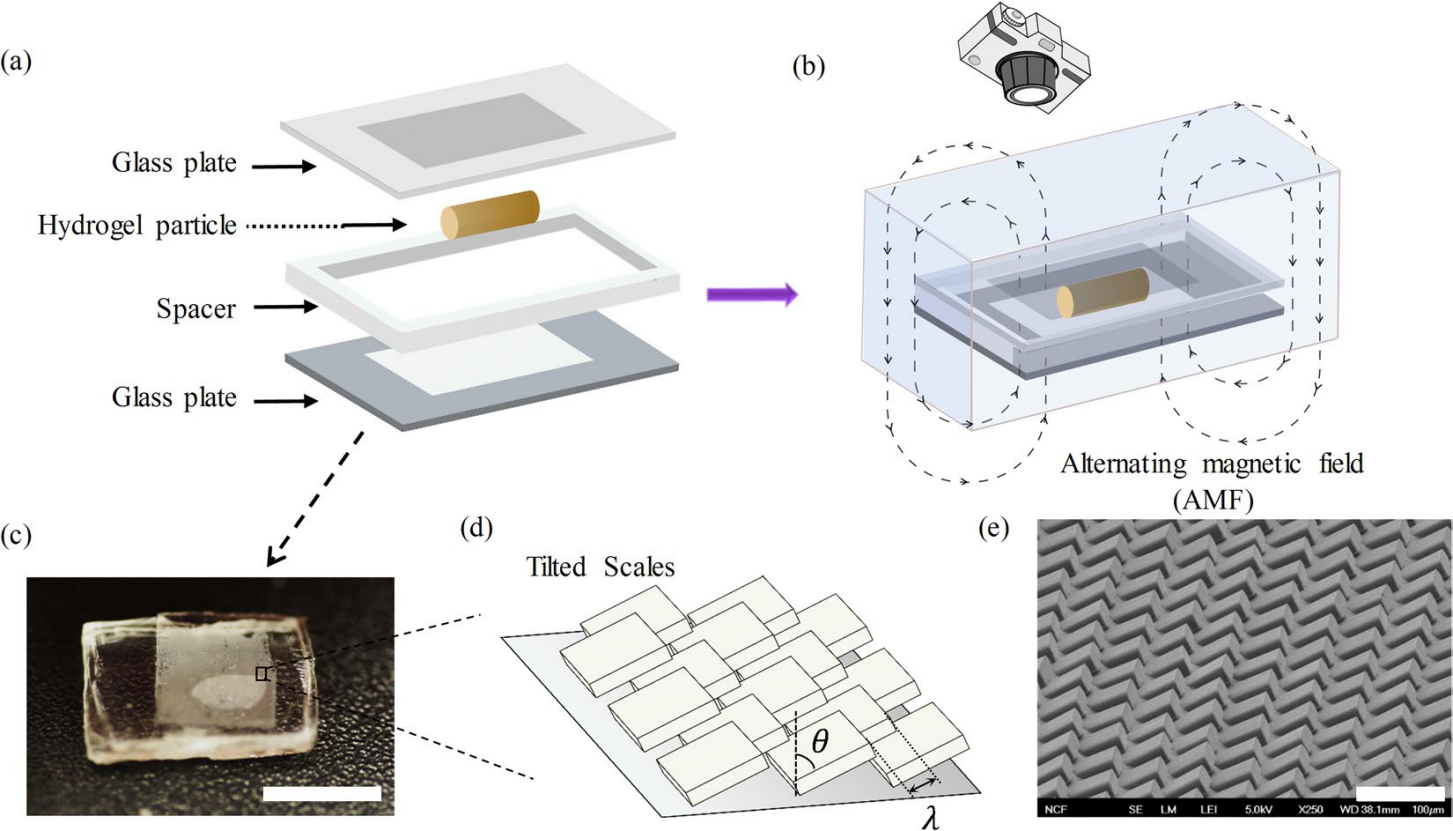
(**a**) Schematic of the assembly of the confined chamber. The glass plates on both top and bottom were coated by the asymmetric micro-scales. The radius of the hydrogel particle was *R* = 0.35 *mm* and the height of the spacer was *h* = 0.4 *mm*. (**b**) Schematic of experimental setup, where the confined chamber was immersed in Tween 20 solution and exposed to an alternating magnetic field (AMF) to achieve heating. (**c**) Micro-patterns coated on the glass plates with the scale bar equal to 5 *mm*. (**d**) Schematic of the geometry of the ratchet-shaped surface micropatterns. The geometry of the patterns are characterized by the tilt angle *θ* and distance *λ*. (**e**) SEM image of the surface micropatterns with the scale bar equal to 100 *μm*.

In Fig. 2a, we show the assembly of the active motile system, which consists of a cylinder of diameter *d* = 0.7 *mm* made of a thermally sensitive hydrogel (PNIPAAm) infused with *Fe*_3_ *O*_4_ superparamagnetic nanoparticles, and placed in a confined chamber made of two glass plates separated by a spacer of height *h* = 0.4*m*. We note that this height ensures that the hydrogel particle is always confined during motion. To induce anisotropy, both the top and the bottom plates were covered with micro-pattern (Fig. 2c–e) resembling the scale of animals such as earthworm and maggots^28^, fabricated with a photolithographic-based microfabrication technique on the silicon wafer with an average height of ~70 *μm*. After fabrication, the patterns were transferred to the glass plates with adhesive tapes. After assembly, the confined chamber was immersed in an aqueous surfactant (Tween 20) solution for lubrication. As shown in Fig. 2d, the structure of the scale-like micro-patterns can be characterized by two parameters: the tilt angle of the scales and the scale density. The tilt angle *θ* is defined by the angle between the pattern and the vertical axis shown in Fig. 2d and accounts for the anisotropy of the scales. Under this definition, *θ* = 45° corresponds to isotropic scales and *θ*~90° corresponds to the most anisotropic structure. The scale density is measured by *L*_0_/*λ* where *λ* is the distance between the two neighboring tilted scales (Fig. 2d) and *L*_0_ is the length of the particle at its swollen state in the confined chamber.

### Magnetic Actuation

The thermo-sensitive Poly(N-isopropylacrylamide) (PNIPAAm) hydrogel is known for its extreme sensitivity to temperature around the lower critical solution temperature (LCST) comprised between 32 °C and 36 °C. Indeed, at the LCST, this polymer displays a reversible phase transition switching its response from hydrophilic to hydrophobic. In other words, below the LCST, the polymer absorbs large amounts of water and is highly swollen, while above the LCST, it dehydrates and loses almost 90% of its volume. The periodic actuation of the PNIPAAm particle can then be achieved by periodically exposing it to an alternating magnetic field (AMF), effectively heating the embedded superparamagnetic *F* *e*_*3*_ *O*_*4*_ nanoparticles via Brownian relaxation and Neel relaxation^38^. Thus, when the AMF is switched on, the hydrogel is heated above its LCST, and shrinks^39,40^, while when switched off, the hydrogel cools down below the LCST and swells back to its original volume.

**Figure 3:**
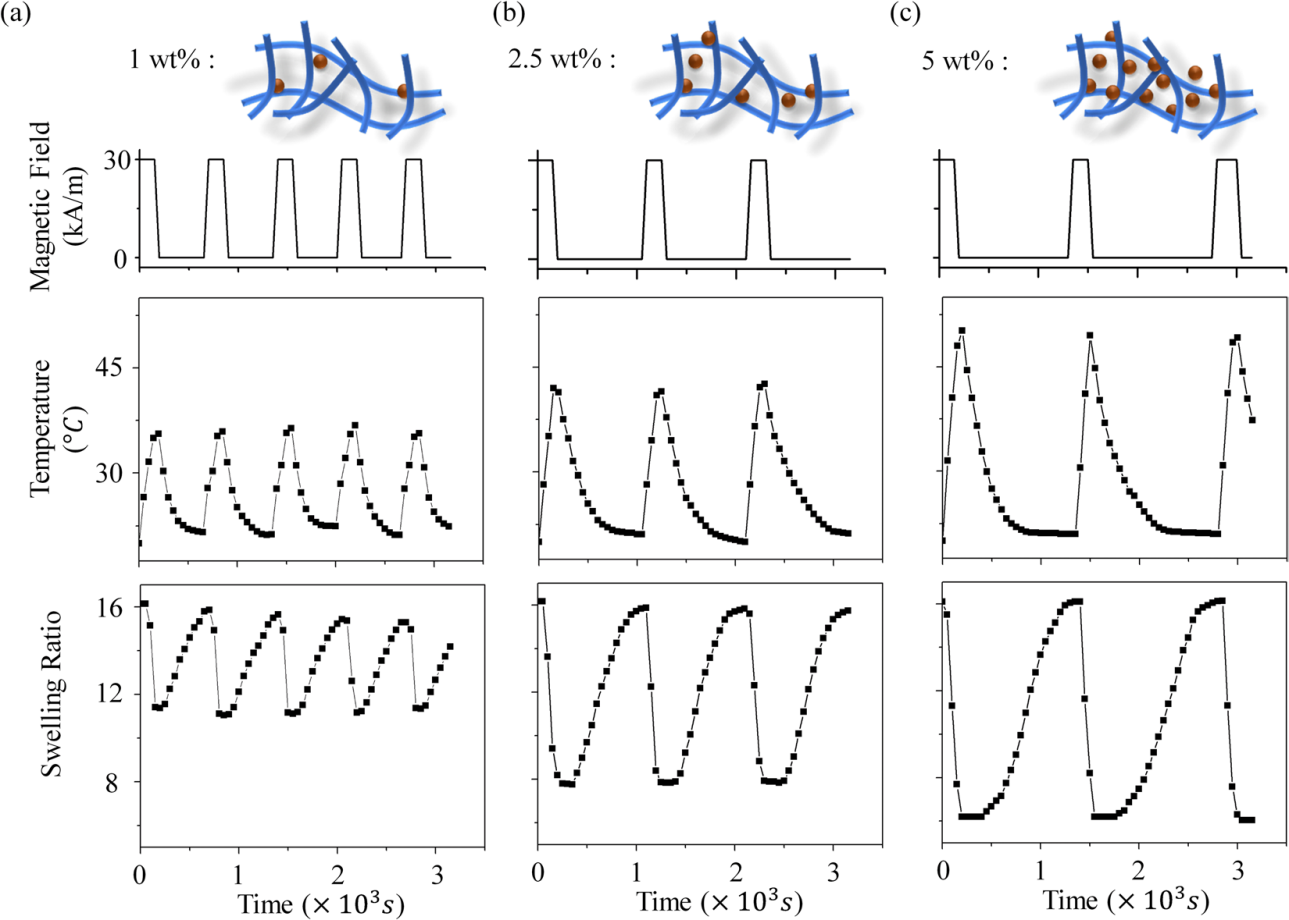
The changes in temperature and swelling ratio of hydrogels with *Fe*_3_ *O*_4_ loadings of (**a**) 1.25 *wt*%; (**b**) 2.5 *wt*% and (**c**) 5 *wt*% in response to AMF stimuli, respectively. In each column, the first row shows the switching on/off of the magnetic field, the second row shows the change in temperature and the third row shows the respective changes in swelling ratio.

To understand how the superparamagnetic nanoparticle concentration affects the hydrogel deformation, we conducted several swelling/deswelling experiments with different *Fe*_3_ *O*_4_ particles loading density (1, 2.5 and 5 *wt*%) under an AMF of frequency *f* = 317 kHz and strength of 30 kA⋅m^−1^. Figure 3 shows the temperature of the particle increased as soon as the AMF was switched on until a constant temperature *T*_*max*_. We observed that the hydrogel particles with higher *Fe*_3_ *O*_4 _loadings had a faster heating rate and higher *T*_*max*_, leading to greater changes in swelling ratio during the heating/cooling cycle. However, the time necessary for cooling was significantly longer at the higher *Fe*_3_ *O*_4_ loading due to the higher *T*_*max*_. In addition, the hydrogel particles exhibited excellent reversible and repeatable contracting/expanding behavior as characterized over 10 heating-cooling cycles, where the volumes at the swollen and unswollen states were varied within 5% and the times for heating and cooling were consistent between cycles. Interested readers are referred to the Supplemental Information for more detailed data.

### Active Motion

In the above discussion of magnetic heating, the hydrogel underwent free swelling/deswelling that was only affected by temperature. However, when the particle is placed in a confined chamber with anisotropic surface scales, its deformation is also affected by resulting frictional forces that act to resist sliding and hydrogel deformation.

**Figure 4:**
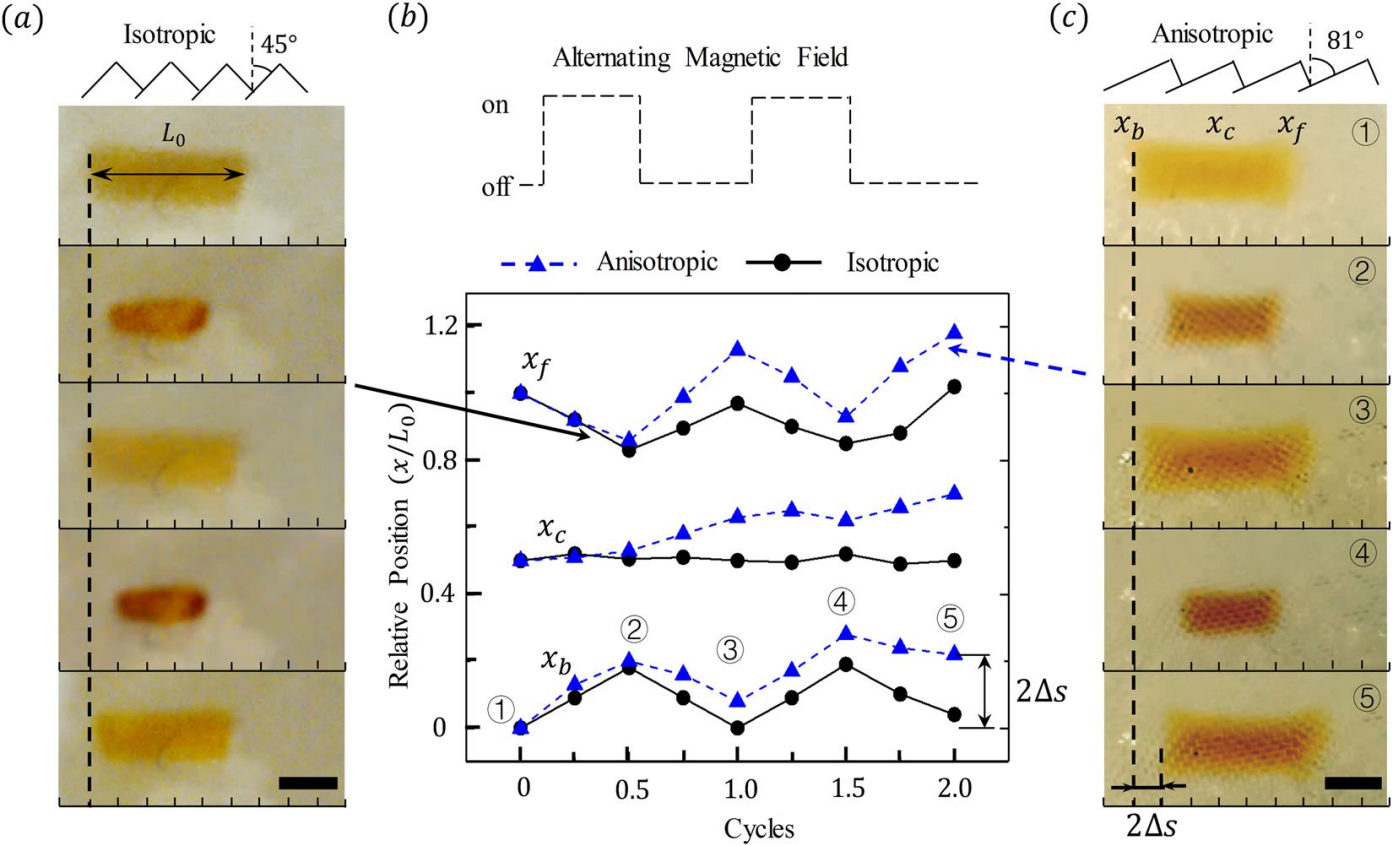
(**a**,**c**) Five sequential snapshots of cyclic deformation of hydrogel crawlers undergoing directional motion, the scale bars equal to 1 *mm*. (**b**) The positions of the front and back of the crawlers, as well as the center positions, over the course of two AMF cycles.

Furthermore, when the hydrogel interacts with the anisotropic scales on the glass surface, the frictional coefficient is different in the forward and backward directions^41^. This feature triggers preferential sliding in the direction of lowest friction and particle motion. For instance, in Fig. 4a,c, we show five sequential snapshots of a hydrogel particle that has been subjected to two deswelling-swelling cycles against surfaces characterized by different scale tilt angles. In Fig. 4a, the scales are isotropic, with tilt angle *θ* = 45°, while in Fig. 4c, the scales are anisotropic, with tilt angle *θ* = 81°.

As a result of the difference in scales, the particle exhibit distinguished mobility on the scales. For isotropic scales (Fig. 4a), the particle gained zero effective locomotion over the course of two cycles. However, for anisotropic scales (Fig. 4c), a clear directional migration towards the right (front) of the particles was observed. To connect this motion to deformation, we show the positions of the front (*x*_f_) and back (*x*_b_) edges of the particles, as well as the position *x*_c_ of their centroid as a function of cycle numbers in Fig. 4c. During the deswelling stages (i.e., ➀-➁), both particles contracted, during which front and back of the particle slid backward and forward, respectively. During the swelling stages (i.e., ➂-➃), the expansion of the particles occurred in a manner that was symmetric to the contraction stage, i.e., the front and back of the particle slid forward and backwards, respectively. In general, we observed that, for isotropic substrate, the forward sliding was equal to the backward sliding in both swelling and deswelling stages, resulting in zero effective motion of the particle centroid; for anisotropic substrate, the forward sliding was greater than the backward sliding for the particles and thus its centroid moved continuously forward, whose step size per cycle is denoted by Δs.

## Discussion

**Figure 5:**
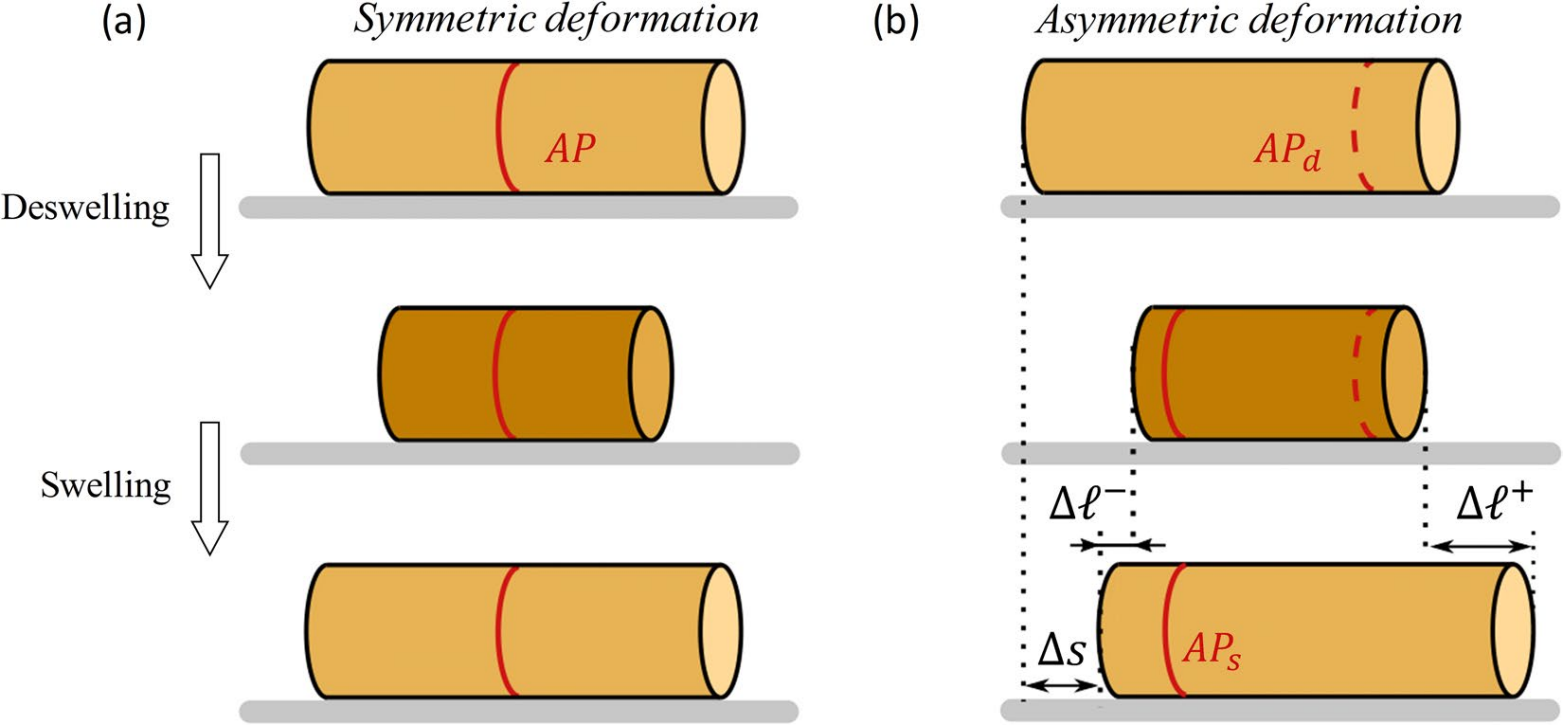
Schematic illustration of a particle that undergoes (**a**) symmetric deformation and (**b**) asymmetric deformation. In (**b**), the position of the anchor point is shifted between the desewlling and swelling stages, denoted as *AP*_*d*_ and *AP*_*s*_, respectively. The displacement of the front and back edges during its elongation is denoted by ${\Delta }{\ell }^{+}$ and ${\Delta }{\ell }^{-}$, respectively.

To better understand the relationship between particle deformation and motion, as well as the dependence of such parameters on the geometry of the micropatterned surfaces, we schematically depict the particle deformation along its principle direction in Fig. 5. It is convenient to introduce the so-called *Anchor Point* (AP), defined as the point on the particle’s surface that displays an effective zero velocity during either the swelling or deswelling state. The location of this point is a hallmark of the substrate asymmetry and motion. Indeed, when the friction properties are symmetric (Fig. 5a), the AP is located at the particle’s center and the deformation is fully symmetric during a deswelling-swelling cycle, yielding a zero effective particle velocity. When the substrate friction is asymmetric, however, a greater portion of particle slides forward, such that the AP is located at the front of the particle during the deswelling stage (AP_d_ in Fig. 5b) and at the back of the particle during the swelling stage (AP_s_ in Fig. 5b). As a result of this biased deformation, the particle continuously moves towards the right with a displacement Δs.

To further elucidate the how the AP is shifted by friction asymmetry and the effect of this on particle’s motion, we consider the mechanics of a long cylindrical particle (length, $\ell$, cross-section, *A*,) elongated in a confined chamber. For this, let us define a coordinate system (0,x) associated with the particle in its undeformed configuration. The longitudinal particle deformation can then be characterized by the displacement *u*(*x*,*t*) and linearized strain *ε* = ∂*u*/∂*x* = *u*_,*x*_ of points along the principle direction. To obtain a qualitative understanding, we use a linear Hookean constitutive law to approximate the mechanical behavior of the particle in the form *σ* = *Eu*_,*x*_ where *σ* and *E* are the stress and Young’s modulus of the gel, respectively. Note that a nonlinear hyperelastic constitutive model can be used for a more accurate characterization of stress-strain relationship as discussed in^34^. The frictional shear stress *τ* between the particle and the patterns is associated with the normal confining pressure *σ*_n_ between them by the friction coefficient *μ* such that *τ* = *μσ*_n_. Again *σ*_n_ may be approximated as constant during particle elongation, which is sufficient for a qualitative understanding of the response, although a thorough investigation of the mechanics in such systems can be found in^34^. Further, the magnitude of *μ* depends on the direction of sliding, where *μ* = *μ* ^+^ if *u*(*x*)  > 0 and *μ* = *μ* ^−^ if *u*(*x*) < 0. Since the gel deformation is driven by osmotic pressure differences between the hydrogel and the surrounding environment in a quasi-static manner^36^, the balance between the elastic and frictional forces leads to the following equation:

1 $$EA{u}_{xx}-2\mu b{\sigma }_{n}=0.$$

Here, *b* is the width of the contact zone between the gel and the patterns. By directly integrating Eq. 1 in the domains on each side of the AP along the particle respectively and ensuring the continuity of strain at the AP, we can obtain a simple relationship between the position of the AP and the deformation of hydrogel as (derivation is provided in Supplemental Information I):

2 $$\alpha =\frac{{\ell }^{+}-{\ell }^{-}}{{\ell }^{+}+{\ell }^{-}}=\frac{1-{\rm{\Delta }}{l}^{-}/{\rm{\Delta }}{l}^{+}}{1+{\rm{\Delta }}{l}^{-}/{\rm{\Delta }}{l}^{+}}$$

where ${\ell }^{+}$ and ${\ell }^{-}$ are the lengths of the domain that moves forward and backward, respectively, (Fig. 5b) and ${\rm{\Delta }}{\ell }^{+}$ and ${\rm{\Delta }}{\ell }^{-}$ are displacements of the front and back edge of the particle, respectively, during the swelling stage, which can be directly measured from experiment.

Besides, using Eq. 1, one can also relate the particle deformation to friction coefficients via the relationship ${\rm{\Delta }}{\ell }^{-}/{\rm{\Delta }}{\ell }^{+}={\mu }^{+}/{\mu }^{-}$ (see derivation in the Supplemental Information I). Therefore, the position of the AP also reveals the anisotropy of friction: for isotropic friction (${\mu }^{+}={\mu }^{-}$), *α* = 0 and the AP is located at the particle centroid, while as friction becomes more anisotropic, the AP is shifted towards the edge of the particle, e.g. ${\mu }^{+} < {\mu }^{-}$ leads to *α* > 0. The motion efficiency can then be evaluated by the displacement of particle centroid over one cycle Δ*s* normalized by its length *L*_0_ which, according to our previous study^34^, is proportional to the deformation anisotropy and the strain. In total, the relationship can be characterized a simple formula that reads:

3 $$\frac{{\rm{\Delta }}s}{{L}_{0}}\propto \alpha \overline{\varepsilon }.$$

where $\overline{\varepsilon }$ is the average strain of the particle. The relationship shown by Eq. 3 tells that there are two ways to control the normalized step size of the particle. Firstly, one can control the asymmetry of its deformation by modifying the anisotropy of friction. Secondly, one can also control the overall deformation (or strain) of the particle.

**Figure 6:**
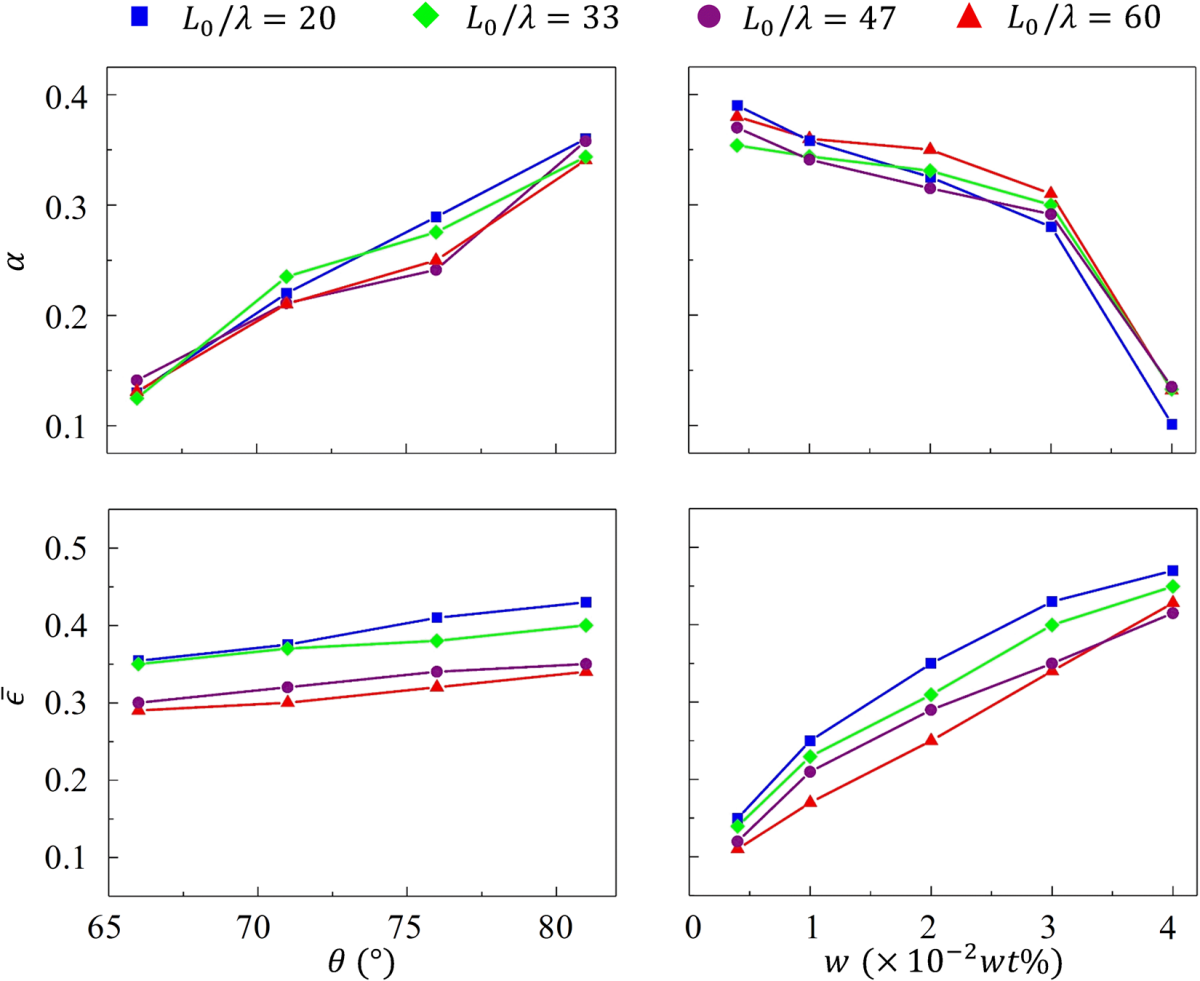
Experimental measurements of particle deformation under different conditions of pattern structure (*θ*, *L*_0_/*λ*) and lubrication (w). The deformation of the particle is characterized in two aspects: the asymmetry of deformation *α* and the overall particle strain $\bar{\epsilon }$. In the left column, the control lubrication condition was *w* = 0.02 *wt*% and in the right column, the control tilt angle was *θ* = 81°.

To test these predictions experimentally, we prepared micro-patterns with different structures, and studied how the hydrogel deformed and moved under different lubrication conditions. Figure 6 shows how the particle deformed, characterized by *α* (calculated by Eq. 2) and $\overline{\varepsilon }$, for different pattern structures in the fluid media with different Tween 20 concentrations *w*. As expected, increasing the tilt angle *θ* led to a more pronounced friction asymmetry and a larger value of *α*. It however had little effect on the strain $\overline{\varepsilon }$, meaning that the overall pattern’s resistance to particle deformation was unchanged by the tilt angle. Interestingly, changes in scales density *L*_0_/*λ* had opposite effects; its role in deformation asymmetry was insignificant while its role on particle strain was significant. Indeed, for higher scale density, the particle was in contact with more patterns during its deformation; this eventually led to a higher frictional stress and a lower strain.

The lubrication condition plays a more intricate role on particle deformation. For small *w*, the particle is in full contact with the scales, leading to large frictional forces and a small overall strain. As *w* increases, a lubrication layer is able to form between the particle and the surface patterns, which tends to decrease the overall friction^42^. As a consequence, the particle is able to achieve larger strains $\overline{\varepsilon }$ but because of the loss of friction, a slight decrease in *α* is also observed. When the fluid media is highly lubricated, the particle loses contact with the patterns and the friction loses its asymmetric nature and becomes dominated by the lubricant viscosity. In these conditions, the particle therefore exhibited large, symmetric deformation, and no translation.

**Figure 7:**
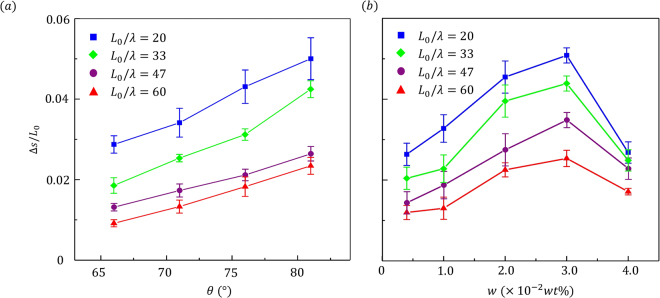
Experimental measurement of particle motion under different conditions of pattern structure (*θ*, *L*_0_/*λ*) and lubrication (w). In the left plot, the control lubrication condition was *w* = 0.02 *wt*% and in the right plot, the control tilt angle was *θ* = 81°.

To complete our study, Fig. 7 shows experimental measurement of particle motion under different conditions of pattern structures (*θ*, *L*_0_/*λ*) and lubrication (*w*). From Fig. 7a, we see that under a given lubrication condition (w = 0.02 wt%), the step size increased monotonically with *θ* while decreased monotonically with *L*_0_/*λ*. As discussed previously, the increase in *θ* indeed favors motion by emphasizing asymmetric deformation while an increase in *L*_0_/*λ* hinders particle sliding by raising frictional forces. The role of lubrication is less intuitive as Fig. 7b shows a nonlinear relation in which neither low nor high lubrication favored motion. At low *w*, the particle indeed achieved asymmetric but small deformation while at high *w*, it exhibited large but symmetric deformation. Therefore, an optimal degree of lubrication can be found as a compromise to these two situations. From these results, it is clear that the step size of the particle can be finely controlled by either modifying the pattern structure or carefully tuning the lubrication condition. We note here that the degree of confinement (represented by the confining pressure *σ*_*n*_ appearing in Eq. 1 can also be acted upon by changing the spacing between the confining plates. We have found in a companion study^34^ that confinement plays a role that akin to lubrication based on similar physical arguments.

## Conclusion

We have presented a remotely actuated system (the mag-bot) that allows a hydrogel particle to actively migrate in confined spaces by utilizing the asymmetric friction generated on microstructured surfaces. Motion is enabled by two key mechanisms: (a) the periodic deswelling and swelling of the hydrogel particle in response to magnetic fields and (b) the asymmetric friction properties enabled by the presence of micropatterns on the substrate. We showed that particle motility in a confined space between two plates strongly depends on both micro pattern-geometry and the level of surface/particle lubrication. These observations were rationalized by simple relationships between the position of the so-called anchor point and the overall strain displayed by the particle during actuation. We found that large geometric asymmetry and low scale concentration favors motion, while the degree of lubrication needs to be carefully tuned to obtain an optimal step size.

Compared to available approaches for self-propelled particles, the possibility of powering motion with remote magnetic fields, not only opens the door to the actuation of multiple crawlers simultaneously, but can also leverage existing technology widely studied in clinical applications^36,37^, Future studies will concentrate on transferring the asymmetric micro-patterns on the soft particle surface, by mimicking similar patterns found on scaled organisms such as maggots, snakes and fish^43–47^. The active deformation of the crawler can also be potentially be improved by considering polymers with reversible networks^48,49^, due to their ability to actively flow, liquid crystal elastomers^50^, or particles that exhibit targeted affinity with their environment^51^. Further studies also need to seek directional control of particles both experimentally and theoretically, using either magnetic or chemical sensing^52,53^. While there is still a long road to go, such particles are promising candidates for remote-controlled delivery vehicles to be used, for instance in targeted drug delivery^1,19,54^, or non-invasive targeted tissue engineering^55–57^.

## Materials and Methods

### Synthesis of Magnetic Hydrogel

N-Isopropylacrylamide (NIPAAm) (700 mM) monomer and N,N’-Methylenebis (acrylamide) (BIS) (8.6 mM) crosslinker were dissolved in deionized water. *Fe*_3_ *O*_4_ (2.5 wt% of monomer solution) was prepared in water and blended with the monomer solution. The mixture was stirred for 15 min to obtain a homogeneous dispersion of the magnetite nanoparticles. The radical initiator Ammonium Persulfate (APS) (0.1 wt% of mixture) and accelerator Tetramethylethylenediamine (TEMED) (2.25 wt% of mixture) were then added and mixed to initiate the redox reaction. After mixing, the solution was injected into a cylindrical mold that was 400 *μm* in diameter and allowed to stand overnight at room temperature to achieve the desired cylindrical iron-encapsulated PNIPAAm hydrogel particles. In order to prevent the particle from sticking to the substrates during experiment, we formed an intercalating lubricating layer by immersing the particles in Tween 20 solution until fully swollen^21^. The diameters of hydrogel particles were 700 *μm* at the swollen state.

The chemicals, including NIPAAm monomer, BIS cross-linker, TEMED, APS were obtained from Sigma Aldrich (St. Louis, MI). The magnetic *Fe*_3_ *O*_4_ nanoparticles (15–30 *nm* diameter) were obtained from US Research Nanomaterials, Inc (Houston, TX).

### Micro-scale substrate fabrication

The microscale ratchet substrates were prepared by utilizing photolithography-based microfabrication technique. All lithographic processes were conducted in the Colorado Nanofabrication Laboratory (CNL) and Joint Institute for Laboratory Astrophysics (JILA) Keck Laboratory at University of Colorado-Boulder. First, photomasks containing microstructures were

fabricated by transferring the structures from transparent masks (CAD/Art Services, Bandon, OR) to chrome (Cr) masks via optical lithography. Specifically, a transparent mask containing microstructures was placed on a Cr mask and was exposed to UV light for 50 sec with a mask aligner (Karl Suss MJB3, Corona, CA). The exposed Cr mask was developed with Microposit

developer concentrate for 40 sec, washed with deionized (DI) water, and dried via nitrogen (*N*_2_) blowing. The Cr mask was then etched with chrome etchant CEP-200 for 70 sec, rinsed with DI water, and dried via *N*_2_ blowing. The remaining photoresist on the Cr mask was removed via sonication for 15 min in photoresist stripper solution AZ 400 T. The Cr mask was then washed with DI water and dried with *N*_2_ blowing.

Next, for fabrication of micro-rectangular pillar structures, 1′′ × 1′′ silicon (Si) substrates were prepared and washed with acetone, IPA and DI water prior to use. The cleaned Si substrates were then blown with *N*_2_ and dried on a hot plate (200 °C for 5 min). The substrates were coated with OmniCoat via spin coating (500 rpm for 5 sec followed by 3000 rpm for 30 sec) and baked at 200 °C for 1 min. The substrates were then coated with roughly 70 *mm* thickness of SU-8 3050 photoresist via spin coating (500 rpm for 10 sec followed by 1700 rpm for 30 sec). The SU-8 3050 coated substrates were baked at 95 °C on a hot plate for 20 min to evaporate residual solvent, exposed to UV light under the photomasks for 35 sec with the mask aligner, and post-exposure baked again at 95 °C on a hot plate for 5 min. These substrates were incubated in SU-8 developer for 18 min, washed with IPA, and dried such that well-defined micro rectangular pillar structures were obtained. By implementing a doctor blading technique, micro-ratchet structures on the Si substrates the micro-pillars were collapsed in a directional manner. These ratchet structures over 1 *cm* × 1 *cm* square areas were then transferred to glass substrates using double-sided tape.

For the materials, Chromium metal and the positive photoresist coated soda lime glasses (i.e., chrome masks) were purchased from Nanofilm (Westlake Village, CA). Microposit developer concentrate was purchased from DOW Electronic Materials (Malborough, MA). Chrome etchant CEP-200 was purchased from HTA Enterprises Microchrome Technology Products (San Jose, CA). Photoresist stripper AZ 400 T was purchased from AZ Electronic Materials USA Corp. (Somerville, NJ). Silicon wafers (100 *mm* diameter, 500 *mm* thickness, single side polished, 100 orientation) were purchased from University Wafer (South Boston, MA). OmniCoat, SU-8 3050 and SU-8 developer were purchased from MicroChem (Westborough, MA). 2-propanol (isopropyl alcohol, IPA) and acetone were purchased from Fisher Chemical (Hampton, NH).

### Magnetic Application

The magnetic heating of the hydrogel was achieved by placing the hydrogel particle in an alternating magnetic field at a frequency of *f* = 317 *kHz*. The magnetic generation set up was built by a high frequency generator (Taylor-Winfield 7.5-135/400-3) with a water-cooling system. In this paper, the magnetic field strength at the center of the coil was controlled as 30 kA m^−1^. The magnetic heating and cooling time were controlled by ensuring that the hydrogel particles reach their unswollen and swollen state during the heating and cooling stage, respectively.

## Electronic supplementary material

Video S1

Supplemental Information

## References

[1] Allen TM, Cullis PR (2004). Drug Delivery Systems: Entering the Mainstream. Science.

[2] Ionov L (2013). Biomimetic Hydrogel-Based Actuating Systems. Adv. Funct. Mater..

[3] Kim S, Laschi C, Trimmer B (2013). Soft robotics: a bioinspired evolution in robotics. Trends Biotechnol..

[4] Yeghiazarian L, Arora H, Nistor V, Montemagno C, Wiesner U (2007). Teaching hydrogels how to move like an earthworm. Soft Matter.

[5] Bergert M, Chandradoss SD, Desai RA, Paluch E (2012). Cell mechanics control rapid transitions between blebs and lamellipodia during migration. Proc. Natl. Acad. Sci..

[6] Friedl P, Weigelin B (2008). Interstitial leukocyte migration and immune function. Nat. Immunol..

[7] Umetani, Y. & Hirose, S. Biomechanical Study of Serpentine Locomotion. In *On Theory and Practice of Robots and Manipulators*, Springer Vienna, 1974, 171–184.

[8] Dreyfus R (2005). Microscopic artificial swimmers. Nature.

[9] Mano N, Heller A (2005). Bioelectrochemical Propulsion. J. Am. Chem. Soc..

[10] Lozano C, ten Hagen B, Löwen H, Bechinger C (2016). Phototaxis of synthetic microswimmers in optical landscapes. Nat. Commun..

[11] Howse JR (2007). Self-Motile Colloidal Particles: From Directed Propulsion to Random Walk. Phys. Rev. Lett..

[12] Izri Z, van der Linden MN, Michelin S, Dauchot O (2014). Self-Propulsion of Pure Water Droplets by Spontaneous Marangoni-Stress-Driven Motion. Phys. Rev. Lett..

[13] Grančič P, Štěpánek F (2011). Active targeting in a random porous medium by chemical swarm robots with secondary chemical signaling. Phys. Rev. E.

[14] Lagubeau G, Le Merrer M, Clanet C, Quéré D (2011). Leidenfrost on a ratchet. Nat. Phys..

[15] Linke H (2006). Self-Propelled Leidenfrost Droplets. Phys. Rev. Lett..

[16] Benet E, Vernerey FJ (2016). Mechanics and stability of vesicles and droplets in confined spaces. Phys. Rev. E.

[17] Benet E, Badran A, Pellegrino J, Vernerey F (2017). The porous media’s effect on the permeation of elastic (soft) particles. J. Membr. Sci..

[18] Foucard LC, Pellegrino J, Vernerey FJ (2014). Particle-Based Moving Interface Method for The Study of the Interaction Between Soft Colloid Particles and Immersed Fibrous Network. CMES Comput. Model. Eng. Sci..

[19] Qiu Y, Park K (2001). Environment-sensitive hydrogels for drug delivery. Adv. Drug Deliv. Rev..

[20] Lendlein A, Shastri VP (2010). Stimuli-Sensitive Polymers. Adv. Mater..

[21] Mahadevan L, Daniel S, Chaudhury MK (2004). Biomimetic ratcheting motion of a soft, slender, sessile gel. Proc. Natl. Acad. Sci..

[22] Lee S-W, Prosser JH, Purohit PK, Lee D (2013). Bioinspired Hygromorphic Actuator Exhibiting Controlled Locomotion. ACS Macro Lett..

[23] Maeda S, Hara Y, Sakai T, Yoshida R, Hashimoto S (2007). Self-Walking Gel. Adv. Mater..

[24] Osada Y, Okuzaki H, Hori H (1992). A polymer gel with electrically driven motility. Nature.

[25] Yang, C. *et al*. Hydrogel Walkers with Electro-Driven Motility for Cargo Transport. *Sci*. *Rep*. 5 (2015). 10.1038/srep13622 PMC4551975 26314786

[26] Morales D, Palleau E, Dickey MD, Velev OD (2014). Electro-actuated hydrogel walkers with dual responsive legs. Soft Matter.

[27] Francis W, Dunne A, Delaney C, Florea L, Diamond D (2017). Spiropyran based hydrogels actuators—Walking in the light. Sens. Actuators B Chem..

[28] Roberts MJ (1971). On the locomotion of cyclorrhaphan maggots (Diptera). J. Nat. Hist..

[29] Heckscher ES, Lockery SR, Doe CQ (2012). Characterization of Drosophila Larval Crawling at the Level of Organism, Segment, and Somatic Body Wall Musculature. J. Neurosci..

[30] Keller JB, Falkovitz MS (1983). Crawling of worms. J. Theor. Biol..

[31] Maeda S, Hara Y, Yoshida R, Hashimoto S (2008). Peristaltic Motion of Polymer Gels. Angew. Chem..

[32] Yoshida R, Ueki T (2014). Evolution of self-oscillating polymer gels as autonomous polymer systems. NPG Asia Mater..

[33] Maeda, S., Kato, T., Takahashi, K. & Hashimoto, S. Active gel locomotion. in *2013 International Symposium on Micro-NanoMechatronics and Human Science (MHS)*, 1–4 (2013).

[34] Vernerey F, Shen T (2017). “The mechanics of hydrogel crawlers in confined environment. J. R. Soc. Interface.

[35] Mura S, Nicolas J, Couvreur P (2013). Stimuli-responsive nanocarriers for drug delivery. Nat. Mater..

[36] Meenach SA, Hilt JZ, Anderson KW (2010). Poly(ethylene glycol)-based magnetic hydrogel nanocomposites for hyperthermia cancer therapy. Acta Biomater..

[37] Hergt R, Dutz S, Müller R, Zeisberger M (2006). Magnetic particle hyperthermia: nanoparticle magnetism and materials development for cancer therapy. J. Phys. Condens. Matter.

[38] Vaishnava PP (2007). Magnetic relaxation and dissipative heating in ferrofluids. J. Appl. Phys..

[39] Afroze F, Nies E, Berghmans H (2000). Phase transitions in the system poly (N-isopropylacrylamide)/water and swelling behaviour of the corresponding networks. J. Mol. Struct..

[40] Cai S, Suo Z (2011). Mechanics and chemical thermodynamics of phase transition in temperature-sensitive hydrogels. J. Mech. Phys. Solids.

[41] Gidoni P, Noselli G, DeSimone A (2014). Crawling on directional surfaces. Int. J. Non-Linear Mech..

[42] Coles JM, Chang DP, Zauscher S (2010). “Molecular mechanisms of aqueous boundary lubrication by mucinous glycoproteins. Curr. Opin. Colloid Interface Sci..

[43] Vernerey FJ, Barthelat F (2010). On the mechanics of fishscale structures. Int. J. Solids Struct..

[44] Vernerey FJ, Barthelat F (2014). Skin and scales of teleost fish: Simple structure but high performance and multiple functions. J. Mech. Phys. Solids.

[45] Funk N (2015). Bioinspired Fabrication and Characterization of a Synthetic Fish Skin for the Protection of Soft Materials. ACS Appl. Mater. Interfaces.

[46] Vernerey FJ, Musiket K, Barthelat F (2014). Mechanics of fish skin: A computational approach for bio-inspired flexible composites. Int. J. Solids Struct..

[47] Marvi, H. & Hu, D. L. Friction enhancement in concertina locomotion of snakes. *J*. *R*. *Soc*. *Interface* rsif20120132 (2012). 10.1098/rsif.2012.0132 PMC3479897 22728386

[48] Vernerey FJ, Long R, Brighenti R (2017). A statistically-based continuum theory for polymers with transient networks. J. Mech. Phys. Solids.

[49] Brighenti, R., & Vernerey, F. J. A simple statistical approach to model the time-dependent response of polymers with reversible cross-links. *Composites Part B: Engineering* **115**, 257–265 (2017). 10.1016/j.compositesb.2016.09.090 PMC5567811 28845123

[50] DeSimone A, Gidoni P, Noselli G (2015). Liquid crystal elastomer strips as soft crawlers. J. Mech. Phys. Solids.

[51] Stefferson MW, Norris SA, Vernerey FJ, Betterton MD, Hough LE (2017). Effects of soft interactions and bound mobility on diffusion in crowded environments: a model of sticky and slippery obstacles. Phys. Biol..

[52] Shen, T. & Vernerey, F. Phoretic motion of soft vesicles and droplets: an XFEM/particle-based numerical solution. *Comput*. *Mech*., 1–19 (2017). 10.1007/s00466-017-1399-y PMC5708599 29200544

[53] Foucard L, Vernerey FJ (2016). A particle-based moving interface method (PMIM) for modeling the large deformation of boundaries in soft matter systems. Int. J. Numer. Methods Eng..

[54] Foucard, L., Espinet, X., Benet, E. & Vernerey, F. J. The Role of the Cortical Membrane in Cell Mechanics: Model and Simulation. *Multiscale Simul*. *Mech*. *Biol*. *Mater*. 241–265 (2013).

[55] Bryant, S. J. & Vernerey, F. J. Programmable Hydrogels for Cell Encapsulation and Neo-Tissue Growth to Enable Personalized Tissue Engineering. *Adv*. *Healthc*. *Mater*. n/a-n/a. 10.1002/adhm.201700605 PMC5826758 28975716

[56] Sridhar SL (2017). Heterogeneity is key to hydrogel-based cartilage tissue regeneration. Soft Matter.

[57] Akalp, U., Bryant, S. J. & Vernerey, F. J. Tuning tissue growth with scaffold degradation in enzyme-sensitive hydrogels: a mathematical model. *Soft Matter* (2016). 10.1039/c6sm00583g PMC5341105 27548744

