# Supplementary material for: Remotely Triggered Locomotion of Hydrogel Mag-bots in Confined Spaces
Source: Sci Rep. 2017 Nov 23;7:16178. doi: 10.1038/s41598-017-16265-w (PMC5701057; doi:10.1038/s41598-017-16265-w)
Supplement: Supplementary file 2 — Supplemental Information [file 41598_2017_16265_MOESM2_ESM.pdf]

Supplemental Information

Remotely Triggered Locomotion of Hydrogel Mag-bots in Confined Spaces

Tong Shen,[a] Marti Garriga. Font,[a] Sukwon Jung,[b] Millicent L. Gabriel,[b] Mark P. Stoykovich [b] and Franck J. Vernerey *[a]

[a] Mechanical Engineering, University of Colorado Boulder, 427 UCB, Boulder, USA.

[b] Chemical and Biological Engineering, University of Colorado Boulder, 596 UCB, Boulder, USA

1. Analysis of magnetic actuation

In the experiment of magnetic actuation of hydrogel, hydrogel particles loaded with different Fe_3O_4 densities $(1, 2.5$ and $5\,wt\%)$ are placed under an AMF of frequency frequency $f = 317$ kHz and strength of $30\ \mathrm{kA \cdot m^{-1}}$ over the course of 10 heating-cooling cycles. In the paper, due to the limitation in space, we only showed several cycles of oscillation in temperature and volume of the hydrogel particles in response to the magnetic field. To complement the study, in Figure 1a, we show the swelling ratios of hydrogel particles at their contraction and expansion states (repentance $N = 3$). We observed that the hydrogel particles with higher Fe_3O_4 loadings have a lower swelling ratio at their unswollen states since they are subjected to higher temperature, as shown in Fig.3 in the paper. However, the effect of Fe_3O_4 loading on the swelling ratio of the swollen state is insignificant. Besides, it is shown that the hydrogel particles exhibited excellent reversible and repeatable contracting/expanding behavior, where swelling ratios at swollen and unswollen states were consistent as characterized over 10 heating-cooling cycles. In Figure 1b, we show the change in volume V_s/V_u of hydrogels during the heating-cooling cycles, where V_s and V_u are their volume at the swollen states and unswollen states. Again, good reversibility is observed and V_s/V_u increases for hydrogel with higher Fe_3O_4 loading.

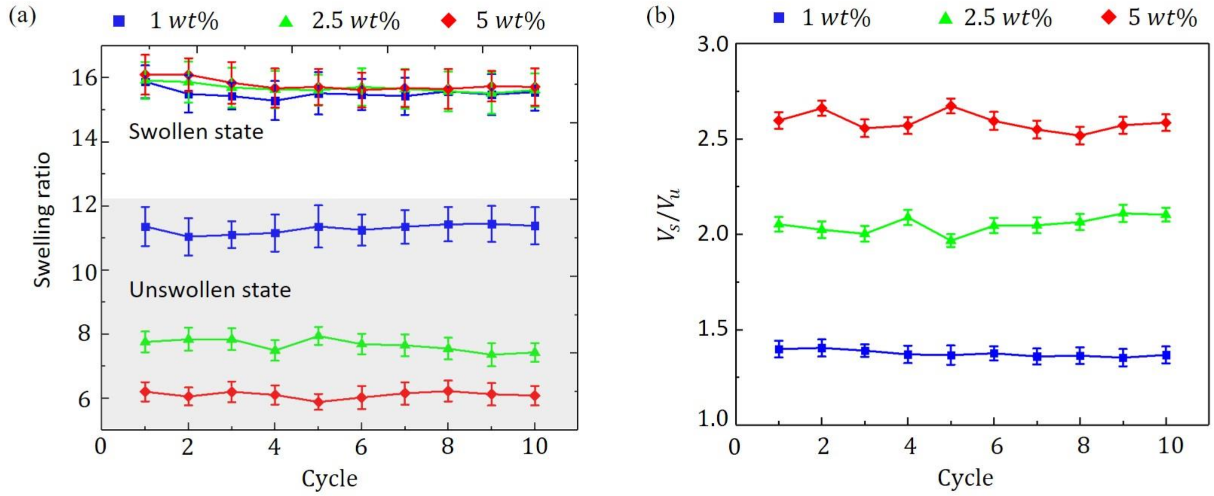

Figure 1 （a） Average swelling ratios of hydrogels loaded with different Fe_3O_4 concentrations at their expansion and contraction states over the course of 10 heating-cooling cycles. (b) The average volumetric expansion V_s/V_u for each Fe_3O_4 concentration during the heating-cooling cycles.

In addition to studying the deformability of hydrogel, Figure 2 summarizes the average time expense for contraction/expansion for hydrogels with different Fe_eO_4 loadings. We find that as the Fe_eO_4 loading increases, although the hydrogel is able to reach its unswollen state faster, the time necessary for it to expand is significantly longer because it possess a higher maximal temperature. As shown in Fig.3 in the paper. In the active motion experiments, to achieve a relatively short time for contracting and expanding while ensuring sufficient change in swelling ratio, we chose to use the hydrogel particle with Fe_3O_4 concentration of 2.5 $wt\%$.

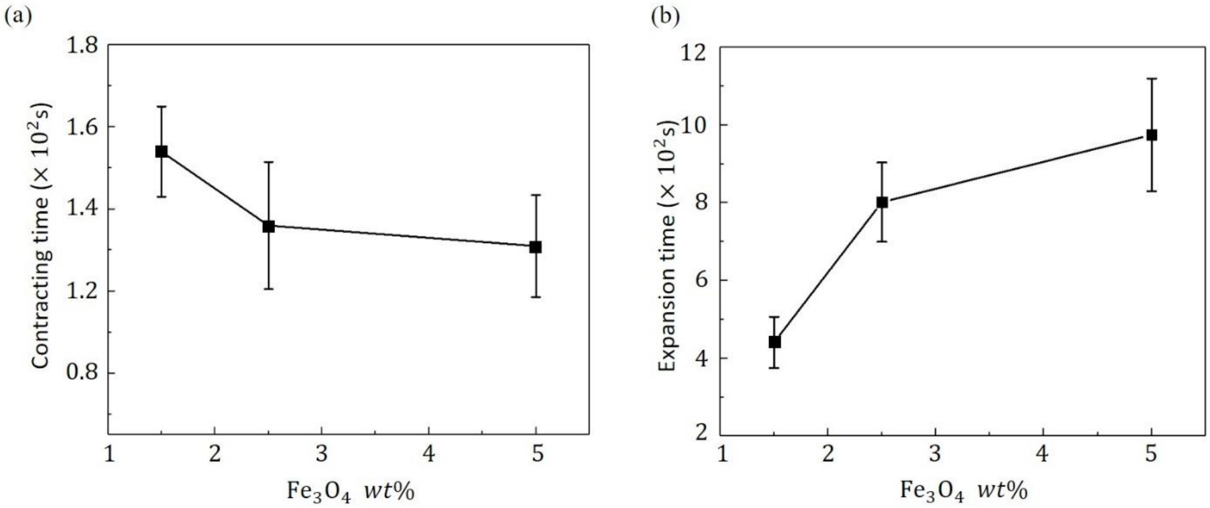

Figure 2. Average time expense for the hydrogel particles to (a) contract from swollen to unswollen state and (b) expand from unswollen to swollen state.

2. Analysis of Mag-bot deformation

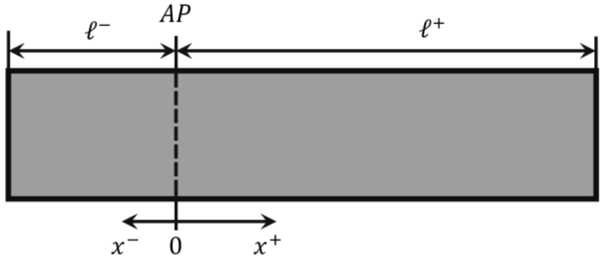

Figure S3. Schematic of the particle and the coordinate systems.

The balance between the elastic and frictional forces of hydrogel deformation leads to Eq.1 written as:

$$EAu_{x,x} - 2\mu b\sigma_n = 0 \tag{S1}$$

with boundary conditions $u_{,x}(x^{\pm} = \ell^{\pm}) = 0$ describing the stress-free conditions at the particles extremities and $u_{,x}^{+}(x^{+} = 0) = u_{,x}^{-}(x^{-} = 0)$ describing the continuity of strain at the AP. By directly integrating Eq.S1 in the domains on each side of the AP along the particle respectively and consider the boundary conditions, one can obtain the linearized strain $u_{,x}(x)$ in each domain as:

$$u_{,x}^{\pm}(x) = \frac{2b}{EA}\mu^{\pm}(\ell^{\pm} - x^{\pm})\bar{\sigma}_n^{\pm} \tag{S2}$$

where $\bar{\sigma}_n^{\pm} = \frac{1}{\ell^{\pm}}\int_0^{\ell^{\pm}} \sigma_n^{\pm}\, dx$ describe the average normal stress of each domain. Here, we use the conclusion of our previous study [1] to relate the lengths of each domain to friction coefficients by: $\mu^{+}\ell^{+} = \mu^{-}\ell^{-}$. Plugging this relation in Eq. S2 and ensuring the continuity of strain at the AP, we can then obtain the following equation:

$$\frac{u_{,x}^{+}}{u_{,x}^{-}} = \frac{\mu^{+}(\ell^{+} - x^{+})}{\mu^{-}(\ell^{-} - x^{-})} \tag{S2}$$

The ratio between displacements of the front and back edge of the particle can then be obtain from Eq. S2 as the following form:

$$\frac{\Delta\ell^{+}}{\Delta\ell^{-}} = \frac{\int_0^{\ell^{+}} u_{,x}^{+}\, dx}{\int_0^{\ell^{-}} u_{,x}^{-}\, dx} = \frac{\ell^{+}}{\ell^{-}}. \tag{S3}$$

In summary, we can find the relationships between the edge displacements, friction coefficients and the lengths of each domain as follows:

$$\frac{\Delta\ell^{+}}{\Delta\ell^{-}} = \frac{\ell^{+}}{\ell^{-}} = \frac{\mu^{-}}{\mu^{+}}. \tag{S4}$$

[1] F. Vernerey and T. Shen, “The mechanics of hydrogel crawlers in confined environment,” *J. R. Soc. Interface*, vol. 14, no. 132, p. 20170242, Jul. 2017.
